# Nearly isotropic piezoresistive response due to charge detour conduction in nanoparticle thin films

**DOI:** 10.1038/srep11939

**Published:** 2015-07-15

**Authors:** Cheng-Wei Jiang, I.-Chih Ni, Shien-Der Tzeng, Watson Kuo

**Affiliations:** 1 Department of Physics, National Chung Hsing University, Taichung 402, Taiwan; 2 Department of Physics, National Dong Hwa University, Hualien 974, Taiwan; 3 Institute of NanoScience and Research Center for Sustainable Energy and Nanotechnology, National Chung Hsing University, Taichung 402, Taiwan

## Abstract

Piezoresistive responses of nanoparticle thin-film strain sensors on flexible polyimide substrates were studied. Disordered interparticle tunneling introduces microscopic detour of charge conduction so as to reduce gauge factors. The disorder also results in large resistance change when current flows in the direction perpendicular to a unidirectional strain, reducing response anisotropy. For practical usages, stability and endurance of these strain sensors are confirmed with 7 × 10^4^ bending cycles. Cracks form in devices under prolonged cyclic bending and slightly reduce gauge factor.

Strain sensors have a long history in their development and applications^1^. Recently the emerging mobile electronics revokes these studies because of the heavily relying on a high-performance and low-cost component in human-machine interfaces^2^,^3^,^4^,^5^. Piezoresistance devices^6^, of which resistance changes according to stress (or strain), are good candidates for such a need. Nowadays piezoresistance devices fall in two categories: the resistance change of metallic ones is due to shape change, and that of semiconducting ones due to resistivity change. In comparison to metallic strain sensors, semiconducting ones may produce 100 times resistance change under the same strain^7^,^8^. Yet the metallic sensors, receiving benefit of low-cost, still have wide applications.

Recently, people have discovered that strain sensors made of metallic nanoparticles (NPs) may have a greater performance, which is comparable to that of semiconducting ones^9^,^10^,^11^,^12^,^13^,^14^,^15^,^16^,^17^. Such an enhancement arises from a strain-sensitive hopping conduction in these NP films based on the conductance formula in weak coupling regime at high temperatures^9^,^18^,


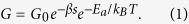


Here *s* is interparticle spacing, while *β* is a constant, typically between 9–13 nm^−1^, depending on free space tunneling or molecule chain hopping as interparticle conduction mechanism^19^. *E*_*a*_ is thermal activation energy that is related to single particle Coulomb charging effect. At room temperature, *E*_*a*_ of NP with a size larger than 10 nm is usually smaller than thermal energy *k*_B_*T* (~26 meV as temperature *T *~ 300 K), and the factor *e*^−*βs*^ affects majorly. Assuming that the organic molecules, which support interparticle spacing *s*, are much more easily deformed than the NPs, we can estimate the change of *s* under an isotropic expansion is given by Δ*s *=* *(2*r* + *s*)*ε*, in which *r* is NP radius and *ε* denotes strain. From Eq. (1), relative resistance change as a function of *ε* reads:


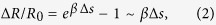


in which *R*_0_ is device resistance in absence of strain, Δ*R *= *R* − *R*_*0*_ the resistance change, and *g* = Δ*R/εR*_*0*_ ∼ *β*(2*r* + *s*) the gauge factor, which is typically used to quantify the performance of a strain sensor. When *r* ≫ *s*, particle radius *r* becomes the dominant factor affecting *g*: a larger *r* would give a higher gauge factor in this ideal model, and have been confirmed in recent works^20^. Another work studied the effect of *β* on *g*^21^. Recently by summarizing several works, people have reported that a thinner substrate gives a larger *g*^22^. However, the experimental *g* is usually lower than the predicted *β*(2*r* + *s*) value by 2- to 3-fold^9^,^10^,^20^. This reduction is universal despite metal volume fraction and interparticle coupling strength. A guess of the possible explanation is the difference between local and macroscopic strain for NPs supported by flexible substrate^21^. Recent experiments on small angle X-ray scattering for monolayer NP films revealed opposite results for various substrates and this local strain issue is under debate^23^,^24^. Farcau and co-workers also found that the dimensionality of NP films has a strong impact on *g*: the confinement of conduction paths gives NP monolayer a larger *g* than multilayers^15^. In a thicker film, second layer NPs may bridge between first layer NPs, and interlayer gaps are less affected by the applied strain. Such a bridging is possible in samples with disorder in *s*, which brings in a conductivity dependent of film thickness^25^. Other works report that crack formation could strongly affect the experimental *g*^26^. However this finding cannot explain the reduction of *g* in monolayer NP film.

One should bear in mind that in these studies samples are always applied to a unidirectional strain and their transverse responses are overlooked. A typical strain sensor has strong anisotropic response under such a strain: The resistance changes most along the strain direction, whereas there is minimal change or no change in perpendicular direction. In general one may define a longitudinal (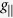) and a transverse (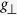) gauge factor to quantify the piezoresistive properties of the NP network. Along the strain direction, stubstrate is elongated by a factor of 1 + *ε* (*ε *> 0), while along the transverse direction, substrate is compressed by a factor of (1 − *νε*). Possion’s ratio, *ν*, reflects the fact that in bulk materials an elongation in one direction usually introduces a contraction in other two principal directions. For silicon substrates, *ν *= 0.22–0.28 while for polyimide substrates, *ν *= 0.33. It can be shown that the two gauge factors obey a simple addition rule, 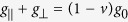 , in which *g*_0_ is the gauge factor under an isotropic strain. A large anisotropy ratio 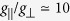 is expected in a triangular NP network when *ν *= 0 (See Supporting Information Sec. I). Grimaldi *et al.* theoretically studied the piezoresistive response and its anisotropy for a bond-percolation model of granular metals. They found that a variation on bond conductance strongly suppressed piezoresistive anisotropy^27^,^28^. For NP networks, the randomness in interparticle tunneling is possible to explain the reduction of gauge factor in the longitudinal direction; it should lead to a large transverse gauge factor as well.

In our experiment, we studied longitudinal and transverse piezoresistive responses of a thin NP film. After studying a large number of devices we found that 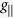 and response anisotropy 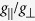 are overestimated. A close inspection reveals that disorder indeed plays an important role in the reduction of 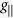 and anisotropy, under the picture – “detour” picture in short – given by Grimaldi *et al.* NP networks would exhibit versatile charge conduction behaviors with disorder, which recently has been explored in a systematically way^29^. Nearly isotropic piezoresistive response renders the NP device a unique strain sensor, a point not discussed before. This isotropic response also elucidates the microscopic charge conduction property in NP networks. We also present stability and durability of our NP strain sensors based on a low-cost fabrication scheme.

## Experimental methods

Gold nanoparticles (AuNPs) of 12 nm in diameter were prepared by sodium citrate reduction of HAuCl_4_, and were further modified by molecules of different lengths^30^; they are 3-mercaptopropionic acid (MPA) and 6-mercaptohexanoic acid (MHA). A centrifugal method^30^ was employed to assemble 2–3 layers of AuNPs onto flexible substrate (50 μm-thick polyimide, PI) with pre-made electrodes. In short, MPA- or MHA-modified AuNP colloidal solution (typically 5 mL, concentration ~3 × 10^12^ NPs/cm^3^) was added to a 30 mL centrifuge tube, together with the (3-aminopropyl)trimethoxysilane (APTMS)-modified PI substrate laid on a polydimethylsiloxane (PDMS) support in the tube. After being centrifuged at 8500 g for 20 min, AuNPs were fully deposited on the substrate, and a multilayer AuNP film were obtained by gently pulling out the sample and drying it in air.

**Figure 1:**
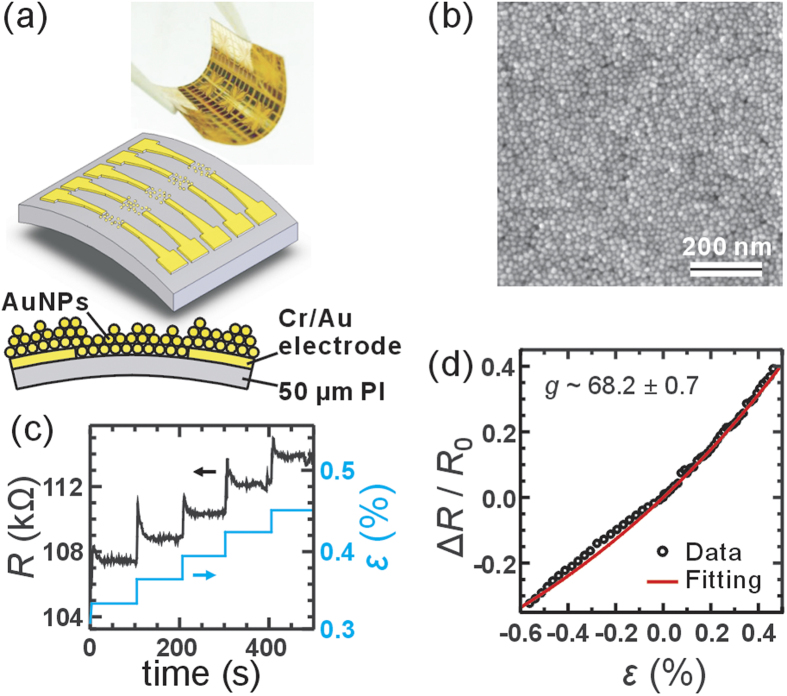
(**a**) A photograph and schematic drawings of the strained AuNP devices. (**b**) SEM image of a typical MPA-modified AuNP film. The film was deposited on a silicon substrate for a clearer SEM image. (**c**) The resistance change of a MPA-AuNP device when the strain was step-by-step increased as indicated by the blue curves. The device presents an overshot in the step response with a characteristic time of about 8 s when the strain *ε* was abruptly changed by ~0.03%. (**d**) The relative resistance change Δ*R*/*R*_0_ as a function of strain from −0.5% to 0.5% (symbols) of the same device corresponds well to the predicted formula 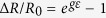 with *g *~ 68.2 (red curve).

Typical device length, *L *= 4–50 μm, was determined by the gap of electrodes. Detail of electrode design can be found in Supporting Information Sec. II. Thermally deposited 20 nm/50 nm Cr/Au electrodes were patterned using e-beam lithography or photolithography, followed by lift-off technique. In prior to the e-beam lithography, large Cr/Au pads and alignment marks were fabricated using photolithography and thermal deposition. For e-beam lithography, double-layer resist, 6% PMMA-co-MAA (Polymethyl-methacrylate-co-methacrylic acid) and 3% PMMA (Polymethylmethacrylate) were used, and exposed with a typical dosage of 50–60 μC/cm^2^. Figure 1a shows a photograph and illustrations of the flexible strain sensor. The AuNP film contains about 2–3 layers of nanoparticles (i.e., thickness = 24 ~ 36 nm), that can be measured by atomic force microscopy (see Supporting Information Sec. III). Figure 1b is the scanning electron microscopy (SEM) image of a typical MPA-AuNP film. Though it was difficult to examine the structure of the AuNPs networks on insulating PI substrate using high-resolution electron microscopy, this examination was done for samples on Si substrates using the same fabrication method, revealing AuNPs in a close-assembled form and well separated by capping molecules (see Supporting Information Sec. IV). The similar sheet resistances of samples on different substrates provided another hint that the quality of NP network was substrate insensitive.

To have a step-by-step change of the strain, sensor substrate was attached (using epoxy glue) onto a printed circuit board, which could be bended on a homemade test platform. Applied strain could range between −0.6% to 0.6% with <0.01% accuracy, which was calibrated by a commercial metal foil strain gauge. Alternatively the sensor substrates could be simply bended and the strain introduced could be determined from radius of curvature *d* and substrate thickness *t* (=50 μm) by 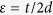. To study the stability and endurance of these sensors, we cyclically bended them to have their radius of curvature *d* changed between 2.80 mm and −3.25 mm, corresponding to *ε *= +0.90% and −0.77% respectively, with a period of 6 sec. All tests were done in ambient condition. The bias voltage for the resistance measurements was 0.1 V, typically. Detail of bending instruments can be found in Supporting Information Sec. V.

## Results and discussions

### The gauge factor

First we study the device response when the strain direction and bias direction are parallel. Figure 1c shows the resistance of an MPA-AuNP strain sensor as a function of time when the strain was increased stepwise as indicated by the blue curves. In this tensile strain test, the resistance *R* overshoots by about 1% and falls to a steady value with a time constant of about 8 s. Overall speaking, the overshooting behavior in a tensile strain is about 10 times larger than that in a compression strain. Besides, the transient behavior in tensile strain is asymmetric while that in compression strain is more symmetric (see Supporting Information Sec. VI). In contrast to the sudden change of strain, a continuous change in strain did not cause significant overshoot comparing to the large resistance change ~30%. Moreover in the stepwise test, we hold the strain for ~100 s for each step to have the device fully relaxed. However in cyclic and continuous tests, strain change only takes seconds so this may partially result in a non-significant overshoot effect. In summary, we believe that the overshoots of the resistance in suddenly strain change are related to the relaxation of the NP film on PI substrate.

These steady values present the sensor response 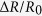 as a function of strain *ε*, as shown in Fig. 1d. More data can be found in Supporting Information Sec. VII. In the small strain region, from −0.5% to 0.5%, we found that 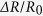 follows the prediction of Eq. (2). Typical commercial metal-film strain sensors have *g* not larger than 5, while our MPA-AuNP films can reach a high gauge factor around 100. Most devices under study fall in the range between 40 and 70, and no clear dependence on device length *L* and surface modification molecule presents, as one can see in Figure S9. Devices on the same chip with similar fabrication process also show variations in *g*. Theoretically, from *r *= 6 nm, *s *= 0.9 nm, and *β *= 11.8 nm^−1^, we would have a gauge factor of about 150. However, in most devices we only observed roughly one-third to one-half of the predicted value, suggesting other effect should be taken into account.

**Figure 2:**
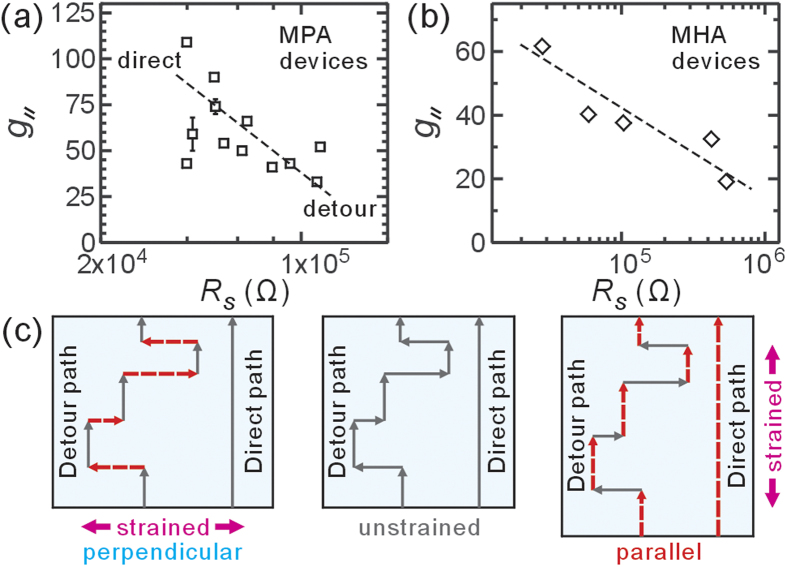
(**a**) The gauge factor 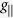 and sheet resistance *R*_*S*_ of 4 μm MPA devices. Because of disorders, devices made of MPA-modified AuNPs would show scattered sheet resistances from 10^4^ to 10^5^ Ω. Intriguingly, the higher device resistance, the lower gauge factor. Error bar in *g* results from fluctuations in various bending tests. (**b**) The same plot for 2-electrode MHA devices shows a similar trend. (**c**) Schematic drawings for the detour scenario: In presence of disorder, the charges conduct through detour paths, which contains both parallel and perpendicular sections so as to bring in higher device resistance than direct path, i.e., *R*_detour _> *R*_direct_. When the device is elongated in parallel direction (Fig. 3c right), the electron hopping in parallel direction greatly reduces while that in perpendicular direction remains the same. This anisotropic change in hopping strength only increases the resistance corresponding to parallel sections (marked by red dash lines). Although the resistance changes in detour and direct paths are similar (i.e., Δ*R*_detour _= Δ*R*_direct_), the larger *R*_detour_ gives a smaller sensor response (i.e., Δ*R*_detour_/*R*_detour _< Δ*R*_direct_/*R*_direct_), resulting in a smaller 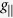. When the device is elongated in perpendicular direction (Fig. 3c left), the perpendicular sections in the detour path give an increased resistance change (Δ*R*_detour _> 0), while the direct path gives zero resistance change (Δ*R*_direct _= 0), resulting in a larger 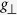. As a result, in the presence of disorder, 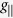 / 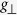
is smaller than the theoretical estimation.

To clarify possible origins of the reduction and variations in 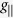, we studied many MPA-AuNP devices, which have a typical sheet resistance *R*_S_ of 10^4^ to 10^5^  Ω, being ideal in practical applications. Besides, the conduction in MPA devices is interesting because they are close to the metal-insulator transition^31^. To find out what factor generates their difference in 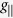, we plot 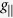 as a function of *R*_S_ for MPA devices, as shown in Fig. 2a. Also to prevent any additional effect arising from electrode geometry, we only present *L*  =  4 μm devices here. Roughly speaking, a higher sheet resistance leads to a lower gauge factor. As pointed in our earlier work, the major effect to result in such a difference in *R*_S_ would be disorder of the NP arrangement^29^,^32^. Indeed one can clearly find disorder in nanoparticle organization in Fig. 1b. Further analysis was done for MHA devices with 2-electrode geometry and is presented in Fig. 2b. Like MPA-devices, these MHA-devices also have large dispersion in gauge factor with roughly one-third of the predicted value. Both groups of device show the similar trend, supporting our proposal on disorder-induced reduction of (parallel) gauge factor. Because the gauge factor of a device related to the microscopic detour of charge conduction, and disorder itself have the nature of large variation in microscopic configuration, one can expect that disorder could produce large variation in the sheet resistance, and the resulting gauge factor also shows large dispersion.

**Figure 3:**
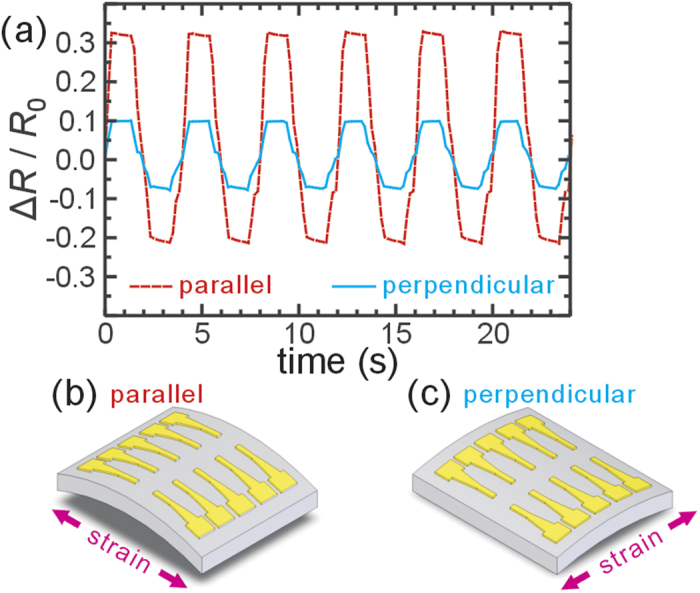
Anisotropic piezoresisitance of the AuNP films. (**a**) The resistance changes of a parallel MHA device and a perpendicular MHA device on the same chip when it was cyclically strained. (**b**–**c**) schematic of parallel and perpendicular configuration of piezoresistance response.

Next, one should notice that these NP devices show an anisotropic response to the unidirectional strain as schematically shown in Fig. 2c. Previous work confirmed that the transverse interparticle spacing generally does not change in monolayer NP film^23^. Figure 3a shows a large perpendicular response for 2-electrode MHA devices than that estimated using our theoretical derivation (also see Supporting Information Sec. I). In general, the anisotropy 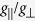 in our devices is surprising low, giving a gauge factor in parallel configuration (Fig. 3b) only slightly larger than that in perpendicular configuration (Fig. 3c).

### The detour picture

The “detour” picture can be understood in an intuitive way for explaining the reduction in 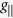 and the anisotropic effect. The disorder in interparticle tunneling hampers electron conduction in such a way that the microscopic charge current no longer flow in the shortest direct path when the electrodes are biased as shown in Fig. 2c. We like to mention that according to Eq. (1), the randomness in *s*, *β* and *E*_*a*_ result in the bond conductance variation considered by Grimaldi *et al.* The first two factors respectively relate to the geometric structure of the NP network and the coverage of capping molecules. The last factor becomes important when the single charge tunneling dominates, i.e., *k*_B_*T * ~  *E*_*a*_. The variation on *E*_*a*_ may arise due to the first two factors as well as the random offset charge on NPs.

The detour causes the actual microscopic route for electron conduction combining both parallel and perpendicular (to the macroscopic current flow direction) sections. Ideally, the parallel section would give a full resistance change, whereas the perpendicular one gives zero resistance change. The macroscopic resistance change would be an ensemble average for these microscopic routes so as to demonstrate a reduced gauge factor. In particular the prominent disorder, the more detours and the smaller gauge factor. Furthermore, we expect as well that the more detour, the longer route an electron conduct from one electrode to another, and larger (sheet) resistance. Since the detour affects both *g* and resistance, we could observe a reduced when *R*_S_ is larger, as shown in Fig. 2a. Also the detour picture asserts that parallel and perpendicular sections coexist in the microscopic routes so as to yield a positive and relatively large 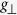.

**Figure 4:**
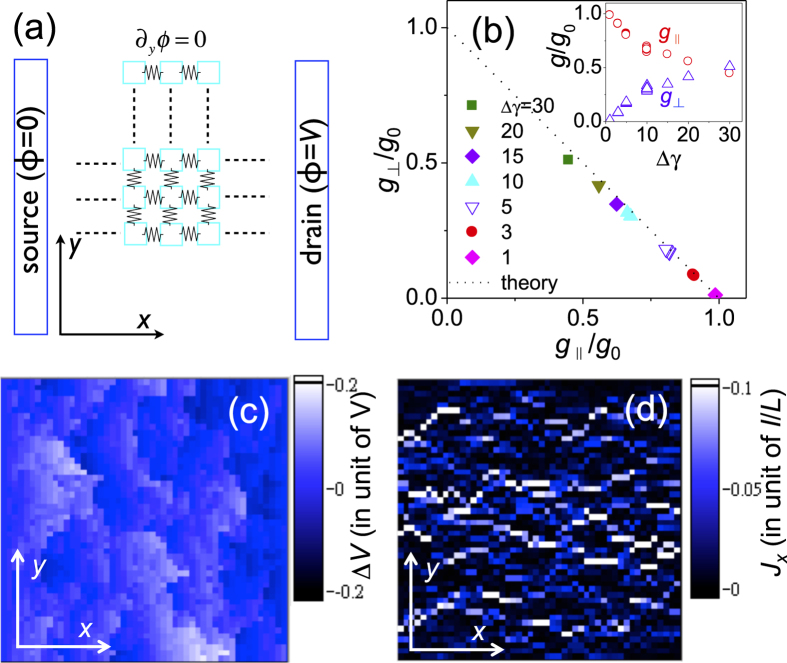
(**a**) The model for numerical calculation. The source (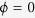) and drain (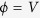) electrodes are placed at *x *= 0 and *x *= *L*. The 2D film is composed of *N *× *N* NPs, which are linked to nearest neighbors with resistors. (**b**) The calculated gauge factors well follow the theoretical prediction 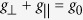. Inset shows how the normalized gauge factors 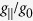 (red) and 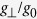 (blue) change as disorder strength 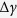 increases. (**c**) The voltage deviation 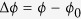, in which 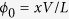 is the homogenous result. The disorder strength is 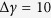. (**d**) A mapping of the current flow illustrates the detour behavior in a disordered 2D system. This configuration gives 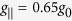 and 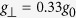.

To gain more understanding on this detour picture, here we introduce a simple theoretical approach encompassing disorder in interparticle tunneling to derive the longitudinal and transverse responses to the unidirectional strain. The two-dimensional (2D) film is modeled by a *N *× *N* matrix of NPs as illustrated in Fig. 4a. Each NP is linked to its nearest neighbors by resistors with a (dimensionless) conductance 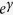, in which *γ* is randomly distributed in the range from 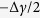 to 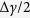. The larger 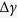 the larger disorder strength. Under the bias voltage *V*, the electric potential *ϕ* in the 2D domain is calculated by current conservation and Ohm’s law (see Supporting Information Sec. IX) subjected to the boundary conditions *ϕ*(*x = *0) = 0, *ϕ*(*x = L*) = *V*, and ∂_y_*ϕ*(*y* = 0,*L*) =0. Here *L* is the sample size. The effects of parallel and perpendicular strain are considered by introducing a small conductance change 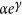 in *x* and *y* directions, respectively. Figure 4b presents the 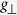 vs. 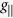 plot for various disorder strength, 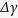. All the data well follow the additional rule 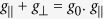 reduces as strength disorder increases, and approaches to the point that 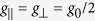 as illustrated in the inset. To illustrate the relation between disorder and detour behavior, we also present the calculation results for a specific configuration of inter-block resistors with 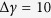: Fig. 4c shows how the voltage deviates from the homogenous solution, while Fig. 4d shows the map of current density in the bias direction, *J*_*x*_ in unit of *I*/*L* with *I* the total current. Clearly one can observe the non-homogenous charge flow meanders through the 2D domain as what we proposed in the detour picture.

### The fatigue test and crack formation

**Figure 5:**
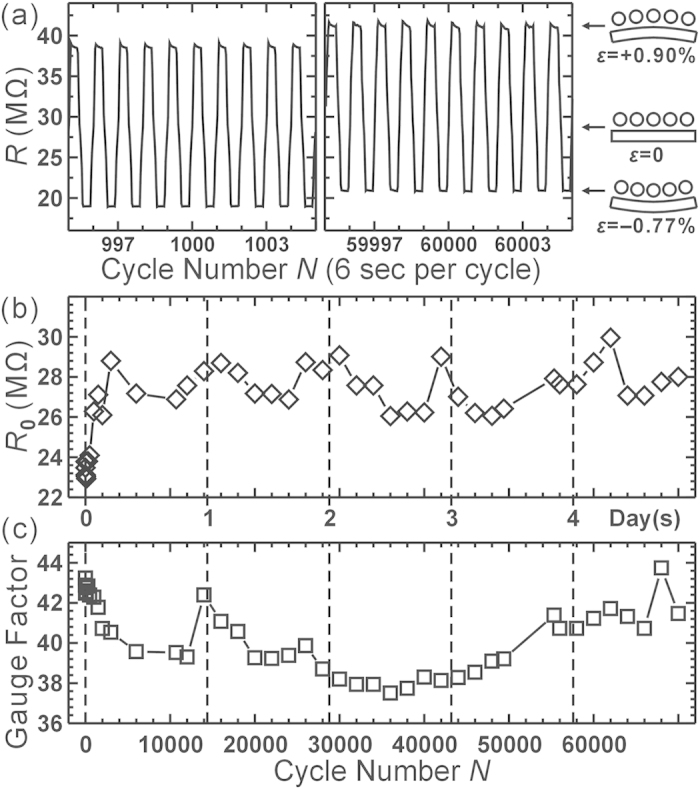
The change of resistance and gauge factor of a MHA sensor while the applied strain cyclically changes between +0.90% and −0.77% with a period of 6 sec. (**a**) the resistance change near cycle number *N *~ 1000 and 60000. (**b**–**c**) The change of *R*_0_ and gauge factor with *N*.

Besides studying what affects the gauge factor of the AuNP strain sensors, examining their stability and endurance are also important for practical applications. To this aim, we performed fatigue tests, repeatedly bent the sensor (6 s per cycle) to have the strain *ε* cyclically change from +0.90% to −0.77%. The testing video can be found in Supporting Information. Figure 5a shows the corresponding change of resistance of a MHA device when the cycle number *N* was about 1000 and 60000. It demonstrates the sensor has good endurance and works well even after bending more than 6 × 10^4^ times. However, *R*_0_ had an obvious increase of about 20% in the first 2000 cycles, as shown in Fig. 5b. This may be caused by the generation of some cracks on the AuNP film. The fatigue test introduced some defects and cracks on the Au/Ni electrodes and AuNP films as illustrated in Figure S9. On the electrodes, long and straight cracks could be as wide as 10 s nm. On the contrary, some isolated defects and small cracks, typically 10 nm in width would be found on the MHA-AuNP films. It has been reported that under strain the cracks become pronounced so that an important factor in strain sensor responses is the possible strain-induced formation of cracks^26^.

However, we only see 10% reduction of gauge factor (from 44 to 40) with the crack formation in our test with *ε *< 1%. After *N *~ 2000, *R*_0_ had a long-term (period ~ 1 day) 4% fluctuation, this may be caused by the variation of humidity in our laboratory under such a long testing time. To clarify this point, we performed the cyclic bending test in humidity and temperature-controlled environment. In stable environment, MPA sensors show monotonic reduction of *g* and increment of *R*_0_ owing to severe cracks formation (cracks about 100 nm wide) during prolonged cyclic bending (Supporting Information Sec. XI). The reason can be understood using the detour picture: Cracks may increase the path of current in perpendicular direction, i.e., increase the detour path, and thus increase the resistance and decrease the gauge factor. Again *g* reduces only by 5%, so we judge that crack formation is not a major effect for the universal *g*-reduction.

On the other hand, we found that by reducing the relative humidity from 55% to 45%, *R*_0_ would decrease about 4.3% (see Supporting Information Sec. XII). This implies the adsorption of water molecules may increase the gap distance *s* between nanoparticles. A resist layer as the device passivation to block the moisture would be a simple remedy for the sensor stability. Figure 5c shows the gauge factor computed from the cyclically change of resistance. It had <8% variation during 7 × 10^4^ cycles (>4 days). These results indicate the sensor has good stability and great endurance for most applications.

## Conclusion

In summary, we used a centrifugal deposition method to build nanoparticle thin-film strain sensors on flexible polyimide substrate. Compared with that of commercial metal film sensors, we found the gauge factors for MPA-AuNP strain sensors are high, ranging from 33 to 109. The disorder in interparticle tunneling plays an important role in the performance of granular metals sensors with soft inter-grain matrix. We picture that the disorder causes microscopic detour paths for charge conduction so as to reduce the gauge factor 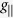. On the other hand, the detour results in resistance change in the transverse direction, resulting in a larger 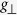 and leads to nearly isotropic response. This isotropic response suggests that “macroscopic” current direction has weak effect on sensor response under a uniaxial strain, giving fewer constrains on electrode design for practical usage. This is very important for practical applications—one can simply ignore the effect from bypassing electrodes and make the large sensor array without isolation. Good stability and endurance of the AuNP strain sensors had also been confirmed with more than 7 × 10^4^ bending cycles.

## Additional Information

**How to cite this article**: Jiang, C.-W. *et al.* Nearly isotropic piezoresistive response due to charge detour conduction in nanoparticle thin films. *Sci. Rep.* **5**, 11939; doi: 10.1038/srep11939 (2015).

## Supplementary Material

Supporting Information

Supplementary video 1

Supplementary video 2
