# Supplementary material for: Nearly isotropic piezoresistive response due to charge detour conduction in nanoparticle thin films
Source: Sci Rep. 2015 Jul 15;5:11939. doi: 10.1038/srep11939 (PMC4502517; doi:10.1038/srep11939)
Supplement: Supporting Information [file srep11939-s1.pdf]

Supporting Information for

Nearly isotropic piezoresistive response due to charge detour conduction in nanoparticle thin films

Cheng-Wei Jiang[a‡], *I-Chih Ni*[b‡], *Shien-Der Tzeng*[b*], *and Watson Kuo*[a*]

[a] Department of Physics, National Chung Hsing University, Taichung 402, Taiwan

[b] Department of Physics, National Dong Hwa University, Hualien 974, Taiwan

‡ Equally contributed to this work

* Corresponding author e-mail:

wkuo@phys.nchu.edu.tw(Kuo), sdtzeng@mail.ndhu.edu.tw(Tzeng)

I. The longitudinal and transverse gauge factors for a trangular NP network.

It is worthy to briefly analyze the anisotropic repsonse of NP monolayer under a unidirectional strain by accounting the irregularity of the NP network. The direction of interparticle tunneling and of the strain would always have an angle θ (Figure S1a). Therefore the parallel and perpendicular projections of interparticle position vector to the strain direction respectively become $a'_{\|} = a\cos\theta(1+\varepsilon)$ and $a'_{\perp} = a\sin\theta(1-\nu\varepsilon)$, in which $a = 2r + s$ is the center-to-center distance of the two neighboring NPs (Figure S1b). Assuming that r does not change under the stress, one has

$$\Delta s = \sqrt{a'_{\|} + a'_{\perp}} - a \simeq \left(\cos^2\theta - \sin^2\theta\nu\right)\varepsilon a \quad .$$

Again because of the randomness of NP network, the tunneling direction and the bias direction in general may have an angle ϕ as shown in Figure S1c. ϕ would vary for different tunneling events, so it is convinient to define a distribution function $P(\phi)$ for describing the macroscopic current flow. For NPs in a triangular lattice, there are 6 nearest neighbors and each neighbor occupies an angle of about π/3 (Figure S1d). An estimation for ϕ distribution function is uniform centered by the bias direction with a cutoff angle $\phi_c \simeq \pi/6$ as shown in Figure S1e. Under this assumption, we calculate the device resistance change as

$$R' \simeq R\int P(\phi)e^{\beta\Delta s}d\phi \,.$$

Therefore when the current direction and strain direction are parallel, namely $\theta = \phi$, the gauge factor reads

$$g_{\parallel} \simeq \beta(2r+s)\int P(\phi)\left(\cos^2\phi - \sin^2\phi\nu\right)d\phi = (0.913 - 0.087\nu)g_0 \,.$$

When the strain direction is perpendicular to the current direction, one has $\theta = \pi/2 - \phi$, and

$$g_{\perp} \simeq \beta(2r+s)\int P(\phi)\left(\sin^2\phi - \cos^2\phi\nu\right)d\phi = (0.087 - 0.913\nu)g_0 \,.$$

Here $g_0 = \beta(2r+s)$ is the gauge factor under an isotropic strain. The assumption ν = 0.3 gives $g_{\parallel} = 0.89g_0$ and $g_{\perp} = -0.19g_0$; a negative resistance change in the perpendicular direction and a large ratio $|g_{\parallel}/g_{\perp}| \approx 5$ is expected. By choosing $\nu = 0$, we could get positive resisitance change in both directions, $g_{\parallel} = 0.91g_0$ and $g_{\perp} = 0.087g_0$, and a large anisotropy ratio $g_{\parallel}/g_{\perp} \simeq 10$ is also expected.

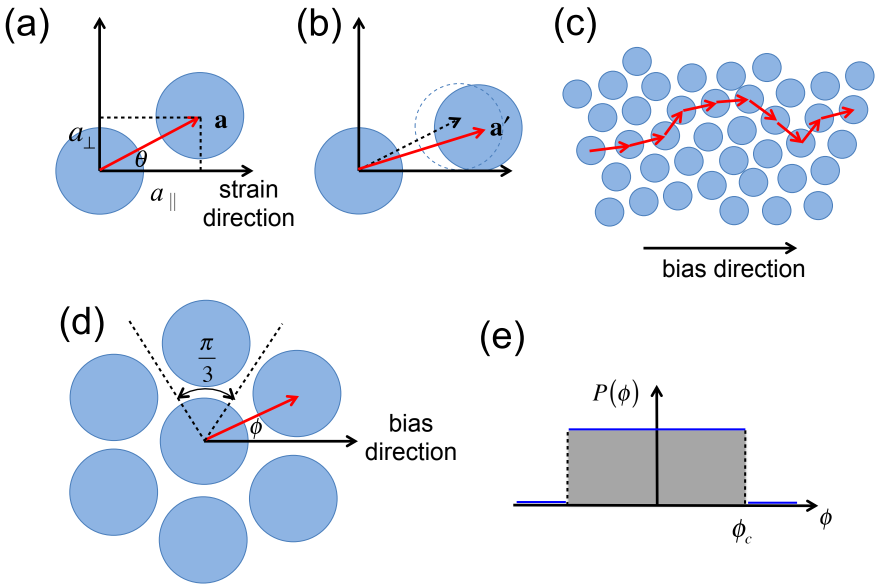

Figure S1. (a) (b) The interparticle displacement vector **a** would have an angle θ to the strain direction. When there is an unidirectional elongation, the displacement vector becomes $\mathbf{a}'$. (c) A schematic describing the sequential charge tunneling through an irrgular NP network. (d) In a triangular lattice, each nearest neighbor NP occupies an angle of about π/3. (e) The assumed uniform tunneling distribution function, $P(\phi)$. $\phi_c \simeq \pi/6$.

II. Device designs

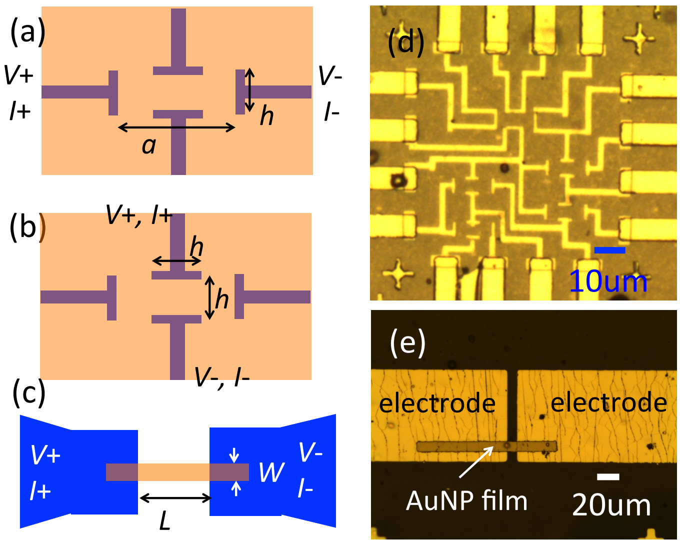

Figure S2. (a)-(c)The designs of AuNP devices. The blue area and orange area mark the electrodes and the AuNP films respectively. (a) "12 μm-devices". When the horizontal pairs were choosen as the measurement leads, the device width is roughly h = 4 μm, while the device length is between a = 12 μm and a-h = 8 μm. (b) "4 μm-device". When the vertical pairs were choosen as the measurement leads, both the device width and device length are roughly h = 4 μm. (c) 2-electrode devices. The device width W is determined by the size of the patterned AuNP film. Typically W = 10 μm. The device length is determined by the gap of the electrodes, L = 5 μm, 10 μm or 50 μm. Large devices with L = 1500 μm and W = 2100 μm were also fabricated. (d) The optical image of "12 μm and 4 μm-devices" on a same chip. We note that the conductivity of the Au electrodes is much higher than that of the AuNP film. Although the NP film covers the whole chip, the small region bounded by the 4 electrodes contributes mostly the device resitance. (e) The optical image of a 10 μm-device. The AuNP film is patterned using optical lithography and standard lift-off technique. Significant cracks form in the electrodes after bending test.

III. Film thickness measured by atomic force microscopy

Thickness of AuNP films was checked by using atomic force microscopy (AFM, Caliber, Veeco Instruments). Figure S3 shows a typical AFM image of a MPA-modified AuNP film. This image was scanned near the edge of a man-made scrape of the film. Its cross-sections show that film thickness is about 35 nm, i.e., about 3 layers of 12-nm nanoparticles.

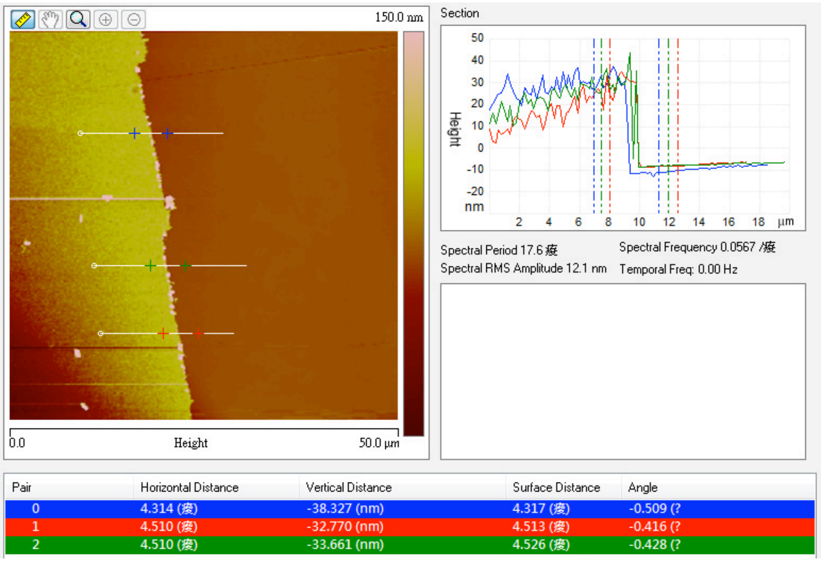

Figure S3. AFM topography image (left) and its cross-sections (right) of a MPA-AuNP film on silicon substrate, taken near the edge of a man-made scrape. The thickness is about 35 nm, i.e., about 3 layers of 12-nm nanoparticles.

IV. Determination of interpartical spacing

Due to insulating PI substrate, it is difficult to get a high resolution image using electron microscopy. Nevertheless, the interparticle spacing can be determined by samples fabricated on silicon substrates using the same deposition methods. In Figure S4(a), we present the transmission electron microscopy (TEM) image of the deposited MUA-AuNPs on carbon coated TEM grid. The interparticle spacing, determined by using the high-resolution images yield a histrogram as illustrated in Figure S4(b), with an average value of 1.9 nm. We note that the resistivity of AuNP films deposited on PI substrate is similar to that of films deposted on silicon substrate. Therefore the interparticle spacing in the two cases should be similar.

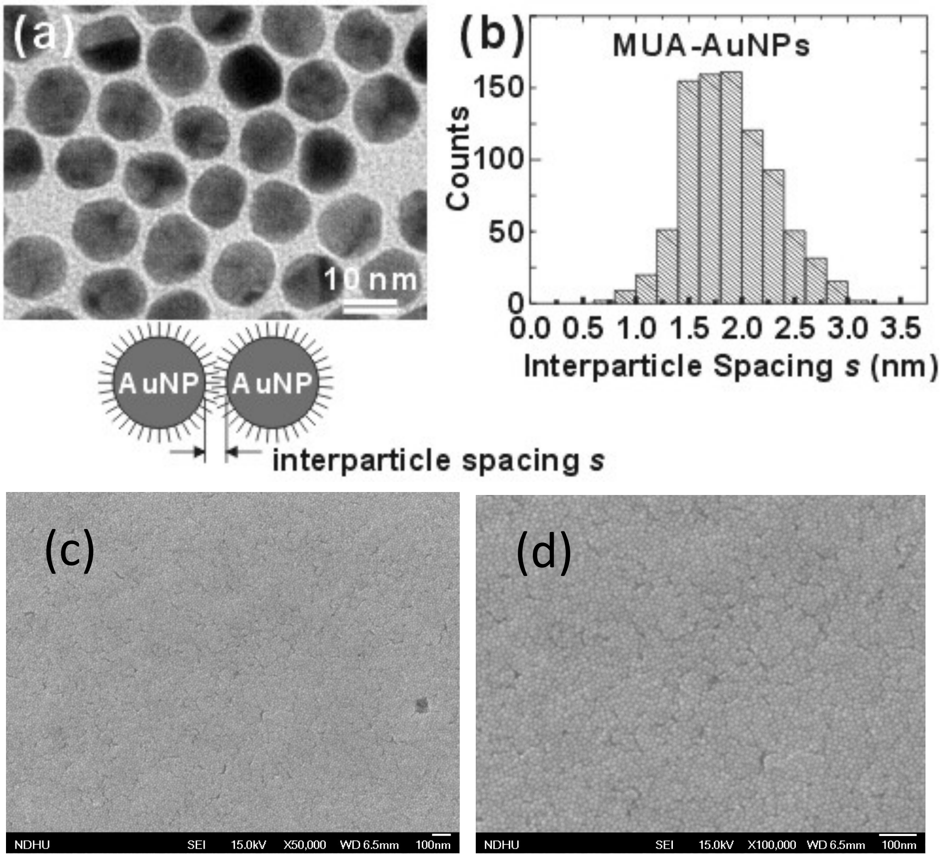

Figure S4. (a) The transmission electron microscopy image of deposited AuNPs modified with MUA molecules. (b) The histrogram of the interparticle spacing for the MUA-AuNP film. (c)(d) The scanning electron microscopy (SEM) images of the AuNP films deposited on PI subsrate. The AuNPs are closely packed to form homogenous film. Both scale bars represent 100 nm.

V. Bending test instrument

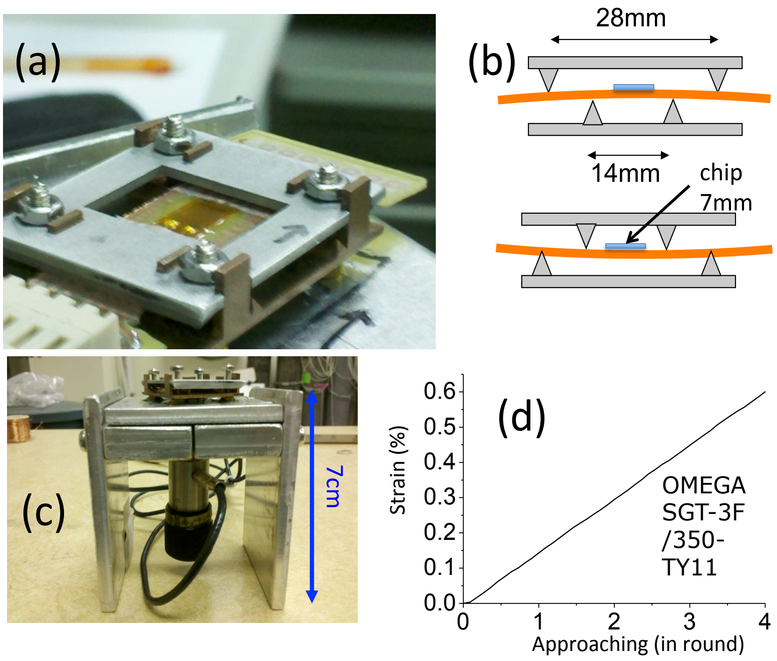

Figure S5. Home-made stepwise bending test platform. (a) Polyimide (PI) chips under test are attached using epoxy glue to a 1 mm-thick printed circuit board (PCB), which is clampped between two metal plates. (b) The clamping plates have wedges spaced by 14 mm and 28 mm respectively. By interchange the two plates, one can change from the elongated strain to the compressive strain. (c) Clamping plates are approached and controlled by a micrometer with a specific approaching distiance, 0.5 mm per round. (d) The strain introduced to device can be calibrated by a commercial metal film gauge sensor (OMEGA, SGT-3F/350 TY11) with a gauge factor of 1.99. 1 round of approaching produces 0.15% strain on the chip.

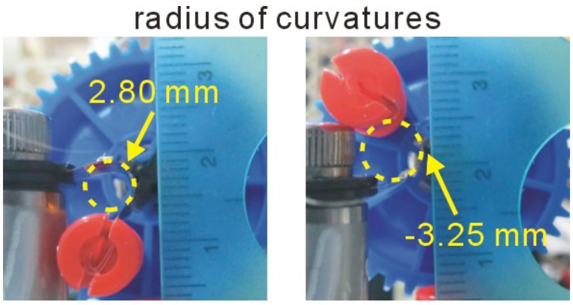

Figure S6. The bending platform for cyclic fatigue test. The radii of curvature for maximal bending are typically 2.80 mm and -3.25 mm, both determined by the pictures.

VI. The device response under an abrupt strain change

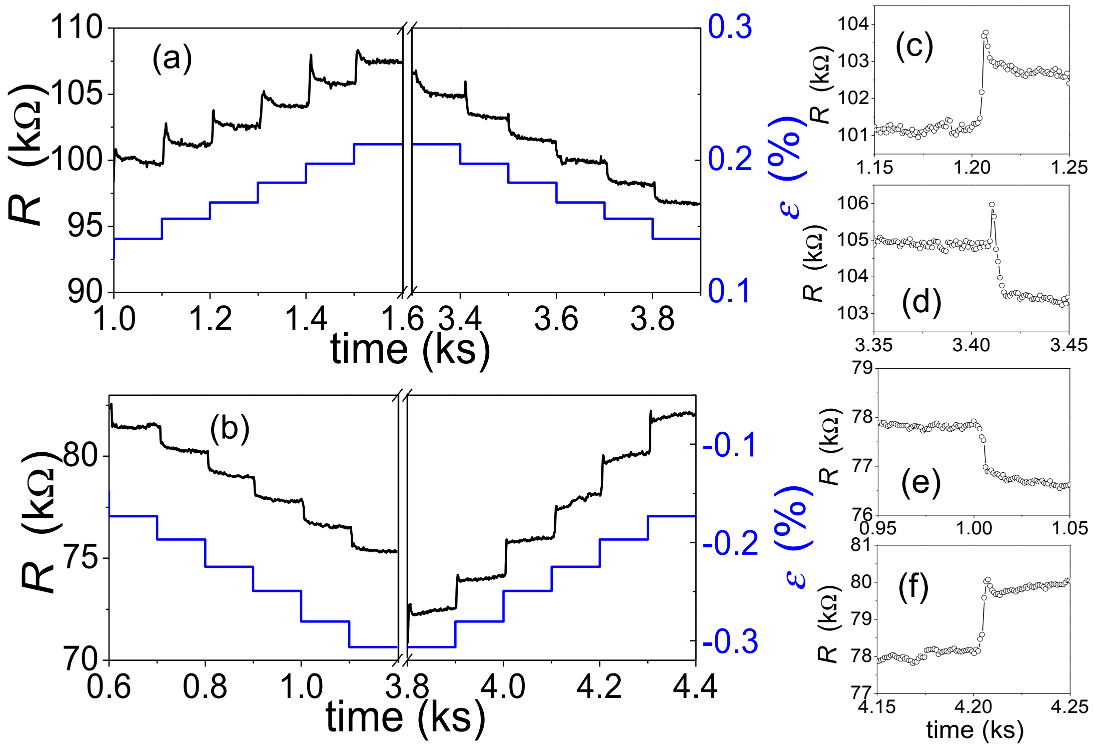

Figure S7. (a-b) The real-time plot of device resisitance when device is stepwisely bended under an elongation (a) and compression (b) strain. The strain increases with time in the first half and releases in the second half. (c-f) The overshooting of the resisitance under an aboupt strain change for increasing (c) and releasing (d) elongation strain, and for increasing (e) and releasing (f) compression strain. The overshooting behavior is large in (c) whilst it is negligible in (e).

VII. Summary of the resistance change under strain

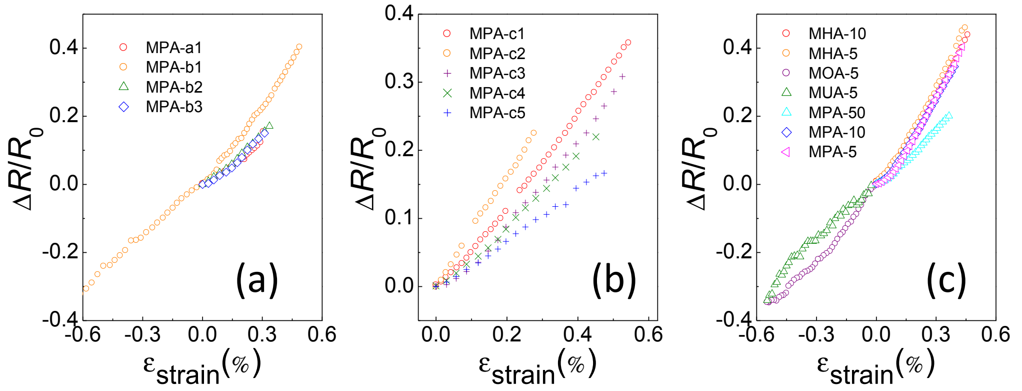

Figure S8. The reisitance change of AuNP devices under a strain. (a) 4 μm (MPA-a1, a2) and 12 μm-devices (MPA-b1, b2) under a parallel strain. (b) 4 μm-devices (MPA-c1, c2) under a parallel strain and 12 μm-devices (MPA-c3, c4, and c5) under a perpendicular strain. (c) 5, 10, and 50 μm-devices with different molecule modifications under the parallel strain.

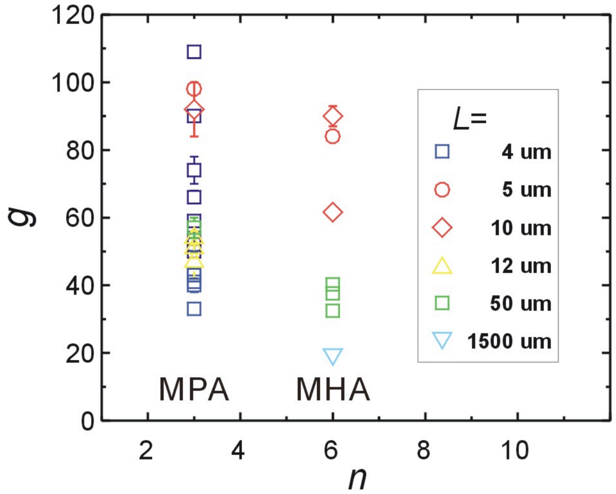

Figure S9. Gauge factors of MPA and MHA devices with different length L (gap distance of electrodes).

VIII. The anisotrpic response

Video named “xy bending test.mov” shows the anisotropic response of the devices under a unidirectional strain. To demonstrate this, we fabricated two strain sensors, whose current flow directions are parallel to the short side (blue device) or long side (red device) of one chip. A multimeter (Keithley 2400, USA) was used to register the device current at a constant voltage bias (0.1 V). The blue device has a larger response when the chip is bended along the short side. On the contrary, the red device repsonses lager when the bending direction is along the long side.

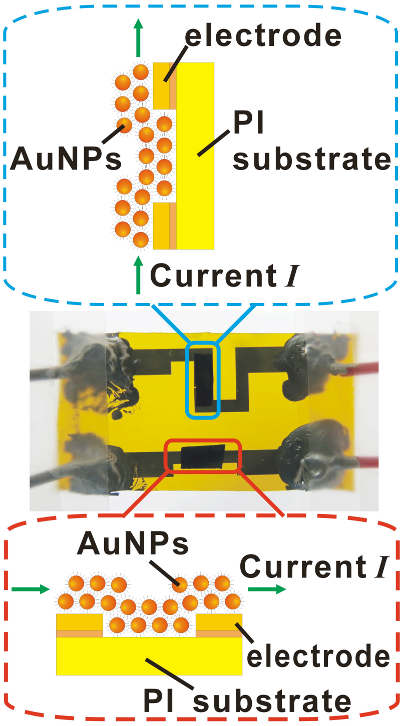

Figure S10. The strain sensors shown in the video named “xy bending test.mov” are MHA-AuNP devices. The width and gap distance of the electrodes are 1 mm and 50 μm, respectively.

IX. Theretical analysis on the detour picture

To gain more understanding on this detour picture, here we introduce a simple theoretical approach encompassing disorder in interparticle tunneling to derive the longitudinal and transverse responses to the unidirectional strain. First, we assume the charge conduction can be fully described by a continuous electric potential, ϕ in two-dimensional (2D) square domain: x, y = 0 — L, with L the sample size. Second, we assume that the charge conduction follows the Ohm's law with a conductivity matrix, σ. In general, the local charge current density follows

$$J_i = -\sigma_{ij}(\nabla\phi)_j$$

with $i(j)$= x and y. In absence of magnetic field, the off-diagonal elements of the conductivity matrix vanish. The current conservation requires that

$$\nabla \cdot J = \partial_i J_i = -\partial_i \sigma_i \partial_i \phi - \sigma_i \partial_i^2 \phi = 0\,. \tag{3}$$

Here we used the contraction convention by dropping the summation symbol $\sum_i$ and used the notation that $\partial/\partial x_i \to \partial_i$ and $\sigma_{ii} \to \sigma_i$.

When there is strain, ε applied in direction-"1" ("1"=x or y), the conductivity changes as follows, $\delta\sigma_1 = g_0\varepsilon\sigma_1 = \alpha\sigma_1$ and $\delta\sigma_2 = 0$. Here for convenience we assume Poisson's ratio $\nu = 0$. The change in the conductivity results in the change in potential, $\delta\phi$ by the current conservation.

$$\delta(\partial_i J_i) = -\delta(\partial_i \sigma_i \partial_i \phi + \sigma_i \partial_i^2 \phi) = 0$$

By using $\delta(\partial_1\sigma_1) = \partial_1\delta\sigma_1 = \alpha\,\partial_1\sigma_1$ and $\delta(\partial_2\sigma_2) = 0$, one obtains

$$\partial_i(\sigma_i \partial_i \delta\phi) = -\alpha\,\partial_1(\sigma_1 \partial_1 \phi)\,. \tag{4}$$

Accordingly, the current change will be

$$\delta J_1 = -\delta\sigma_1 \partial_1 \phi - \sigma_1 \partial_1 \delta\phi = -\alpha\sigma_1 \partial_1 \phi - \sigma_1 \partial_1 \delta\phi \tag{5.a}$$

$$\delta J_2 = -\sigma_2 \partial_2 \delta\phi \tag{5.b}$$

If the 2D film is biased in the x-direction, a parallel current change by assuming the strain in x-direction presents, ("1"=x in Eq. (5a))

$$\delta J_{\parallel} = -\alpha\sigma_x \partial_x \phi - \sigma_x \partial_x \delta\phi_{\parallel}\;.$$

The (perpendicular) current change with strain in y-direction will be ("2"=x in Eq. (5b))

$$\delta J_{\perp} = -\sigma_x \partial_x \delta\phi_{\perp}\,.$$

Here $\delta\phi_{\parallel}$ and $\delta\phi_{\perp}$ are the potential change due to the strain in x and y directions, respectively. From Eq. (4), it is easy to find that they behave in a reciprocal way,

$\partial_i\left[\sigma_i\,\partial_i\left(\delta\phi_\perp+\delta\phi_{||}\right)\right]=-\alpha\,\partial_i\left(\sigma_i\,\partial_i\phi\right)=0$. Because the potential changes obey the boundary conditions $\delta\phi\left(x=0,L\right)=0$ and $\partial_y\delta\phi\left(y=0,L\right)=0$, one can expect $\delta\phi_\perp+\delta\phi_{||}\approx 0$. Furthermore one can assert that

$$\delta J_{||}+\delta J_\perp=-\alpha\sigma_x\,\partial_x\phi-\sigma_x\,\partial_x\left(\delta\phi_{||}+\delta\phi_\perp\right)\approx-\alpha\sigma_x\,\partial_x\phi\,,$$

which leads to

$$g_{||}+g_\perp=\frac{\int\left(\delta J_{||}+\delta J_\perp\right)dy}{\varepsilon\int J\,dy}=g_0\,, \qquad (6)$$

by noting $J=-\sigma_x\,\partial_x\phi$. This is the additional rule mentioned before.

In the homogenous case that $\sigma_x=\sigma_y$ and $\partial_x\sigma_x=\partial_y\sigma_y=0$, the potential follows the Laplace equation $\partial_i^2\phi=0$. The gauge factors are $g_{||}=\delta J_{||}/\varepsilon J=g_0$ and $g_\perp=\delta J_\perp/\varepsilon J=0$, as can be intuitively expected. For the disorder case we employed numerical calculations by assuming different strength of disorder and the results are described in the maintext.

X. Real time response and fatigue test

In video named “cyclic bending test.mov” we present the testing results of our MHA-AuNP strain sensors using a homemade automatic cyclic testing platform. The bending sequences were fully programed for giving static and time-varying strain to the device, and a multimeter was applied as the readout of real time responses. In the first part the device undergoes a continuous cyclic bending using the full range, its response repeats nicely with this fast bending sequences. In the second part, we arbitrarily stopped the bending motion to check the sensor response under a static strain. The response of the device itself shows small transient and drift. Sometimes there are transient-like responses due to the backlash of the power transmission system.

The fatigue test introduced some defects and cracks on the Au/Ni electrodes and AuNP films as illustrated in Figure S11. On the electrodes, long and straight cracks could be as wide as 10s nm. On the contrary, some isolated defects and small cracks, typically 10 nm in width would be found on the AuNP films.

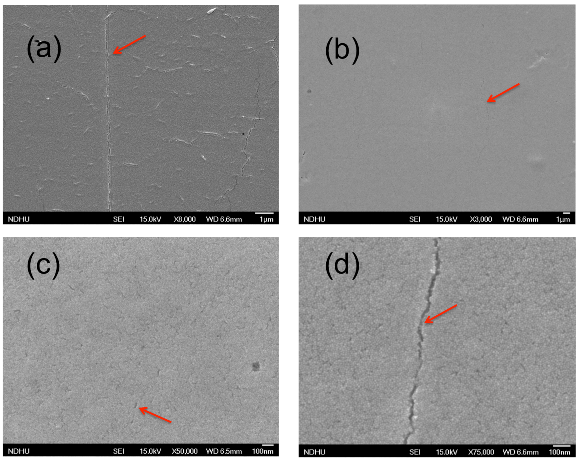

Figure S11. The scanning electron microscopy images of the MHA devices after the fatigue test. (a) On Au/Ni thin film electrode, long cracks with 10s nm in width and 100s μm in length could be found. (b) Much less cracks were found in the AuNP film. (c) On the AuNP film, the large strain may result in some isolated defects. (d) Cracks on AuNP films are much smaller in size, typically 10 nm in width.

XI. Cyclic bending test of MPA device under environment control

A MPA strain sensor and the bending platform (as Fig. S6) were placed in a polymethylmethacrylate (PMMA) box to provide a stable environment (humidity and temperature) for cyclic fatigue test. The relative humidity in the box was 53.5 ± 0.3 % and temperature was 23.9 ± 0.5 °C while performing the test. In contrast to the results of MHA devices (Fig. 5), R_0 (resistance in absence of strain) of the MPA sensor slowly increase with the bending cycle number N. Meanwhile, the gauge factor of the MPA sensor slowly decrease. We supposed that such behavior was related to the formation of cracks in the MPA-AuMP film. As can be seen in Figure S12(c) and S12(d), many cracks formed after N = 20000. These cracks may increase the path of current in perpendicular direction, i.e., increase the detour path, and thus increase the resistance and meanwhile decrease the gauge factor.

Such cracks effect was not obvious for MHA devices (such as results in Fig. 5). We believe it is because the longer MHA molecules between AuNP network have stronger binding energy than the shorter MPA molecules. Thus, MHA-AuNP films have much lower probability of crack formation, as can be seen in Fig. S11(b).

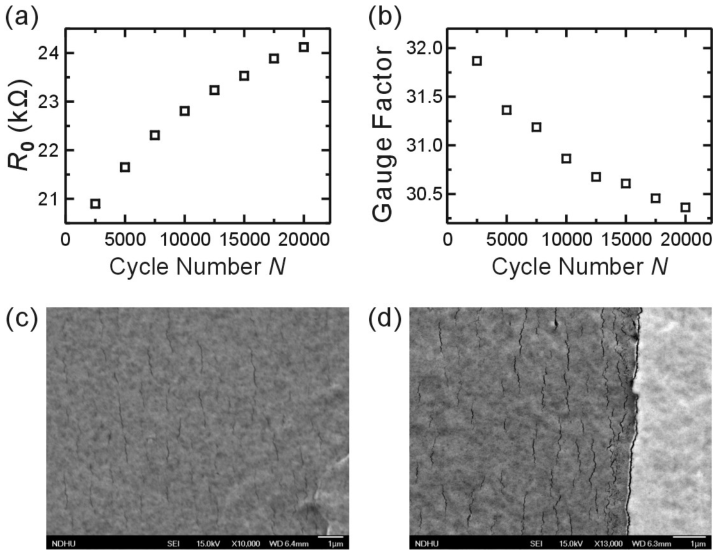

Figure S12. (a) The change of R_0 (resistance in absence of strain) and (b) the change of gauge factor of a MPA-device while the applied strain cyclically changes between +0.75% and -0.83% with a period of 4 sec. The width of the film W = 2 mm, the gap of the electrodes L = 1.5 mm, and the voltage bias is 10 mV. The surrounding relative humidity was 53.5 ± 0.3 % and temperature was 23.9 ± 0.5 °C. (c) (d) The scanning

electron microscopy images of the MPA sensor after bending cycle number N = 20000. (c) Near the edge of Au/Cr thin film electrode, long cracks of the MPA-AuNP film with 10s nm in width could be found. (b) Cracks of the MPA-AuNP film were found far from the edge of electrode.

XII. Humidity dependence

The humidity of the environment may affect the zero-strain resistance (R_0) of the MHA-AuNP device. We found that by reducing the relative humidity from 55% to 45%, R_0 decreased about 4.2%, as shown in Figure S13.

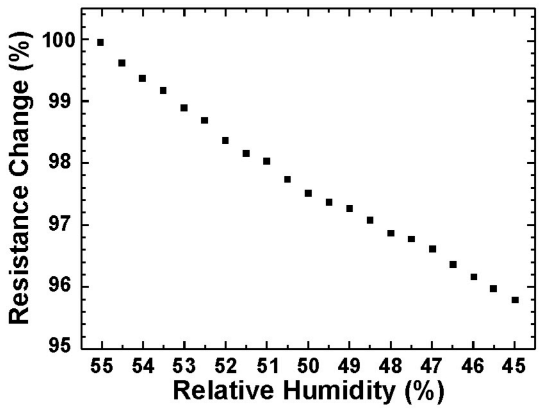

Figure S13. The change of R_0 of a MHA-AuNP device. When the relative humidity changes from 55% to 45%, R_0 decreased about 4.2%.
